# Supplementary material for: Green Extraction Method: Microwave-Assisted Water Extraction Followed by HILIC-HRMS Analysis to Quantify Hydrophilic Compounds in Plants
Source: Metabolites. 2025 Mar 25;15(4):223. doi: 10.3390/metabo15040223 (PMC12029008; doi:10.3390/metabo15040223)
Supplement: Supplementary file 1 [file metabolites-15-00223-s001.zip › metabolites-3525898-supplementary.pdf]

(A) *Vitis Vinifera*

	VV_1	VV_2	VV_3	RSD %
Asparagine	150281	164069	164245	5
Glutamic_acid	721400	811163	792138	6
Glutamine	1940268	2208449	2322827	9
Histidine	61593	78256	85447	16
Leucine	175507	210523	225247	13
Methionine	4907	7126	6882	19
OH-proline	608246	691849	696424	7
Phenylalanine	57314	63951	63795	6
Proline	1659553	1955824	2101166	12
Serine	243542	280725	290321	9.1
Threonine	132490	159597	159328	10
Tryptophan	9159	5606	8346	24
Tyramine	22242	24856	26525	9
Tyrosine	11716	12444	10794	7
Ascorbic_acid	58493	83791	75549	18
Aspartic_acid	305536	375255	392837	13
Gluconic_acid	1576290	1617060	1733517	5
Succinic_acid	147664	157416	158278	4
Fructose	360984	388245	443273	11
Sucrose	18711897	18880336	19198038	2

(B) *Arabidopsis*

	AA_1	AA_2	AA_3	RSD %
Arginine	32190	28840	37978	14
Asparagine	645766	505116	681195	15
Glutamic acid	2275495	1687531	2162312	15
Glutamine	2803204	2390401	2884401	10
Isoleucine	13979	11206	11656	12
Leucine	16187	18477	18188	7
Methionine	8406	7117	9349	14
Phenylalanine	12365	11095	13462	10
Proline	16008	19180	15594	12
Serine	412594	304380	327853	16
Threonine	387318	256773	316606	20
Tryptophan	4759	4543	6095	16
Tyrosine	18212	15892	16663	7
Valine	11198	16697	11742	23
Ascorbic acid	49070	43130	59425	16
Aspartic acid	1936805	1539814	1977524	13
Gluconic_acid	872594	656171	925669	17
Salicylic acid	16554	19853	15994	12
Succinic acid	109003	196506	258195	15
Fructose	564539	620757	496974	11
Glucose	133410	122465	108261	10
Sucrose	1489429	1667688	1178625	17

Additional file S 1: Analysis of the relative standard deviation (RSD) of the polar metabolites in **(A)***Vitis Vinifera* and **(B)** *Arabidopsis* leaves after MAE experimentation. **Three biological replicate** and RSDs were calculated based on area measurements of each compound.

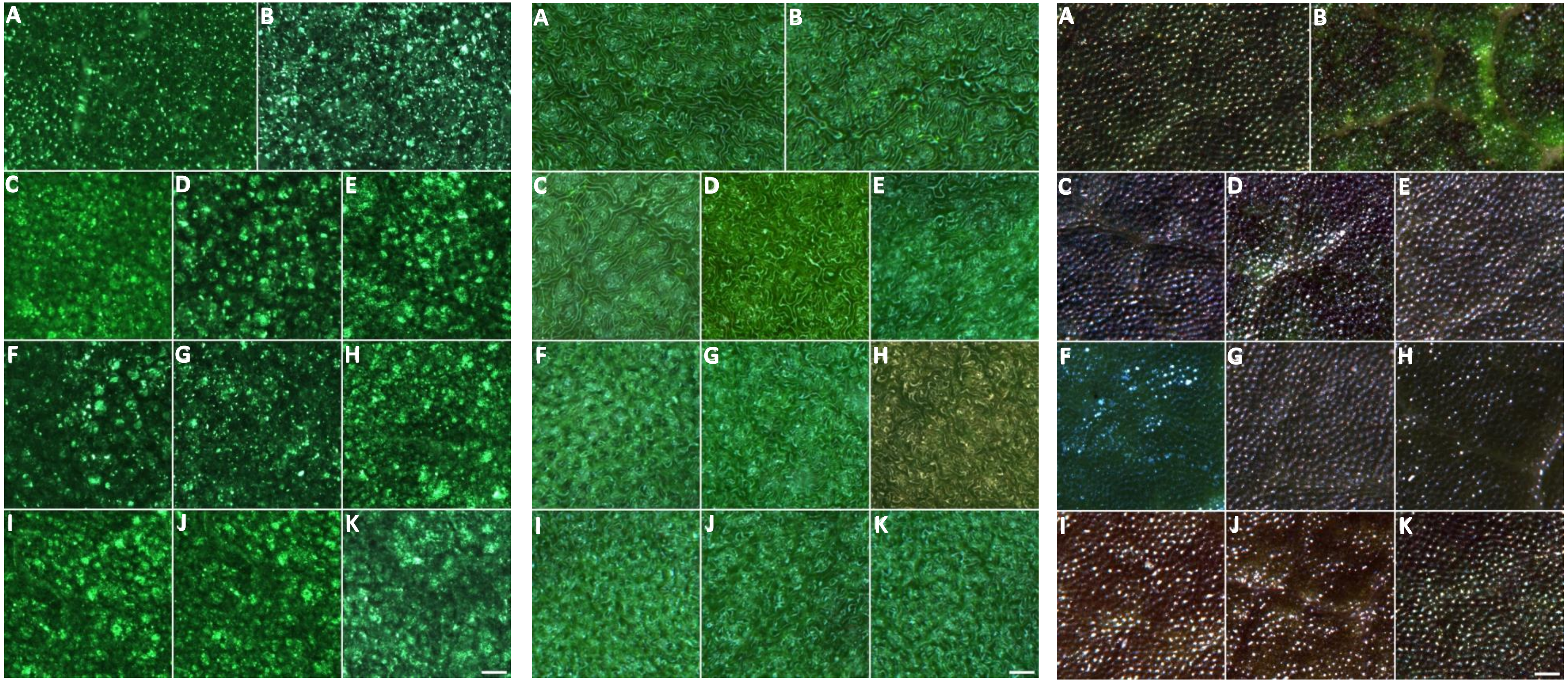

N. benthamiana Arabidopsis Grapevine

Additional file S2: Ultrastructural effects of sonication or microwave treatments on *N. benthamiana*, Arabidopsis and *Vitis vinifera* leaves. For each plant, treatment was identical; MAE was performed with 200 µl of water. A : control; B : sonication 10 mn; From C to K, MAE was performed at the indicated power for various times. C : 300 W, 15 sec; D : 300 W, 20 sec; E : 300 W, 30 sec; F : 450 W, 15 sec; G : 450 W, 20 sec; H : 450 W, 30 sec; I : 600 W, 15 sec; J : 600 W, 20 sec; K : 600 W, 30. Bars represent 100 µm.

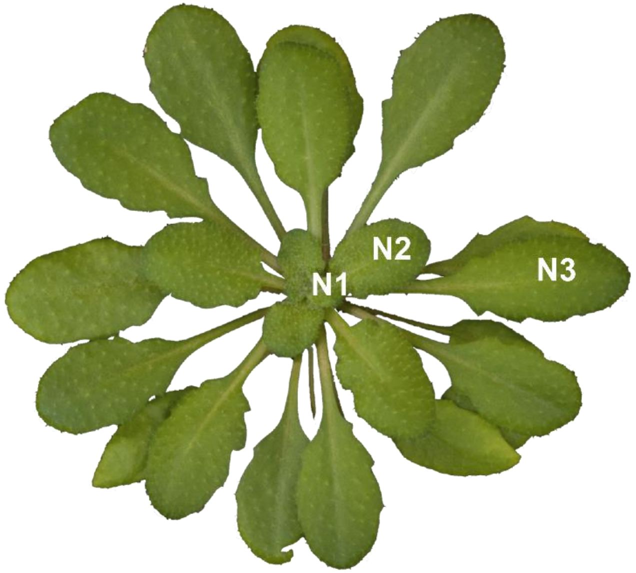

Additional file S3 : Selected developmental stages (N1,N2 and N3) of *Arabidopsis thaliana* rosette leaves.

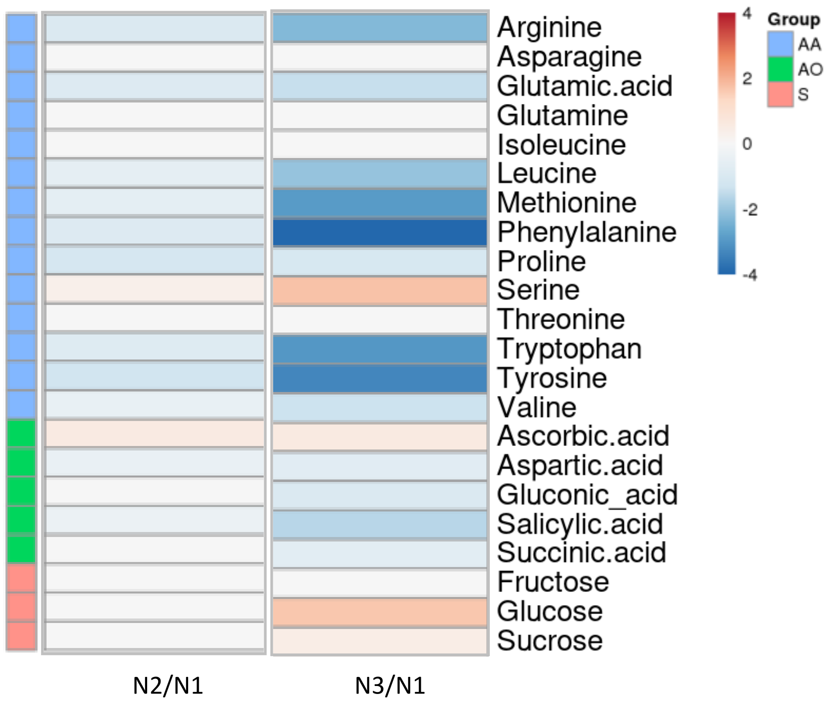

Additional file S4 : Pairwise comparison of changes between distinct spatiotemporal stages (N1, N2 and N3) in Arabidopsis rosette leaves after performing MAE in water. Log2 of significant metabolite fold changes for indicated pairwise comparisons are given by shades of red , white or blue colors according to the scale bar. Data represent mean values of seven to nine biological replicates for each condition and time point. Statistical analysis was performed using Tukey's Honest Significant Difference method followed by a false discovery rate (FDR) correction, with FDR < 0.05. For FDR ≥ 0.05, Log2 fold change was set to 0. The values range from low (blue) to high (red) metabolite-relative content compared to the youngest leaf (N1).
